# The Ethics of Experimentation upon Living Animals

**Published:** 1891-04

**Authors:** 


### The Ethics of Experimentation upon Living Animals.

Cruelty may be defined as the infliction of suffering without sufficient reason. To the humane is an expression of the highest type of manhood. And surely less inhumanity can be traced through the records of science, than can be found in the annuals of either religion or philosophy. Who is more altruistic, more practically philanthropic, than a physician of the better class in his daily intercourse with suffering mankind? Yet these men are periodically assailed by well-meaning if not well-informed persons, and stigmatized as wontonly cruel for performing what, in the majority of instances, are but painless operations upon otherwise useless animals. An action is good, bad or indifferent, in direct ratio to its consequences. Now, those best able to judge admit that vivisection furthers the progress of scientific investigation, and, therefore, concede its defensibility. At the same time, when merely a matter of idle curiosity, it is held to be most reprehensible. Frequent repetitions of painless experiments, however, finds its justification in the necessity for objective instruction. No person will deny that science has conferred incalculable good upon mankind. Nay, more, the burdens of the lower animals are daily being lifted by this modern offspring of the human intellect. Were it not possible to specify an individual discovery directly due to vivisection, nevertheless the fact would remain that the crucial test of all hypotheses is experimentation. And it was not until verification became the anchor of all research that knowledge ceased to drift hither and thither upon the treacherous sea of speculation. But one of the most important steps in the advancement of physiological inquiry, the discovery of the inhibitory function of the pneumogastric nerve by Weber, was the result of an experiment upon a living animal, and it could have been demonstrated in no other manner. Morever, this same method, in the hands of Pasteur, has bestowed sufficient benefit upon the animals themselves to more than compensate for the suffering inflicted upon them in the interest of science.

Unbridled Nature is the personification of cruelty, and the few victims of scientific investigation that are sacrificed for the wellfare of man are as nothing compared to the multitude that meet with apparently purposeless destruction. Certain facts are beyond the reach of the physiologist, except through these experiments; but since, in many instances, pain is a disturbing element, its abolition may be relied upon from this, if from no higher motive. The highest order of man feels unwilling to inflict needless suffer, ing upon any living creature. How much right we have to the lives of the lower animals remains an open question. And thought at this stage of our civilization, it seems to be the general opinion, at least as expressed by man, that the animal shall be slain for our needs, still the causing of unnecessary pain is justly reprobat- ed. Nearly all vivisections, assuredly in our country, are performed after the animals have been rendered insensible; bu- where sentiment blindly forms convictions, reason appears to hold no sway, hence, despite the facts of the case, these perennial crusades against a very necessary method of experimentation. Ultra-humanitarianism in its opposition to scientific advancement is liable to lapse into insipid sentimentality.—Dr. Stephen S. Burt in *The Post-Graduate.*

